# Supplementary material for: Genome-Wide Identification of NAC Gene Family Members of Tree Peony (Paeonia suffruticosa Andrews) and Their Expression under Heat and Waterlogging Stress
Source: Int J Mol Sci. 2024 Aug 28;25(17):9312. doi: 10.3390/ijms25179312 (PMC11395581; doi:10.3390/ijms25179312)
Supplement: Supplementary file 1 [file ijms-25-09312-s001.zip › Table S2.pdf]

Table S2. Basic information and physicochemical properties of 48 NAC genes in tree peony

Gene name	Number of amino acids	Molecular weight (kDa)	Isoelectric point	Instability index	Aliphatic index	Grand average of hydropathicity	Subcellular localization
PsNAC01	565	63368.76	4.67	45.9	62.62	-0.763	N
PsNAC02	299	34087.95	7.43	52.21	57.06	-0.985	N
PsNAC03	255	29715.85	6.36	43.94	75.76	-0.629	Cy, E
PsNAC04	154	17961.74	9.65	31.69	74.03	-0.599	M
PsNAC05	299	34653.90	6.47	48.19	60.64	-0.846	N, Cy
PsNAC06	273	30964.91	5.08	44.98	77.22	-0.610	N
PsNAC07	284	32483.25	8.58	44.09	75.18	-0.376	Cy, Ch
PsNAC08	256	28771.27	9.13	47.38	75.04	-0.418	N
PsNAC09	556	64216.76	4.70	46.32	66.46	-0.858	N
PsNAC10	597	67955.26	6.28	42.14	67.79	-0.691	N
PsNAC11	268	30547.62	8.79	44.74	62.20	-0.711	N
PsNAC12	301	34234.98	6.41	47.93	78.31	-0.510	Cy
PsNAC13	489	53894.86	6.82	52.41	63.64	-0.599	N
PsNAC14	377	43498.38	5.16	49.02	65.44	-0.779	N
PsNAC15	263	29948.97	8.94	40.27	64.07	-0.733	N, Cy
PsNAC16	305	34964.91	8.38	46.10	70.92	-0.470	N, Cy
PsNAC17	243	27204.54	6.00	49.76	62.63	-0.574	N
PsNAC18	438	48529.75	9.31	57.14	66.58	-0.535	N
PsNAC19	337	37145.72	8.89	51.67	57.36	-0.523	N
PsNAC20	162	18699.29	9.23	45.82	63.70	-0.648	N
PsNAC21	288	33103.31	6.01	43.36	72.74	-0.594	N
PsNAC22	326	37487.78	5.78	39.21	56.81	-0.862	N
PsNAC23	410	46299.39	5.07	47.45	67.20	-0.869	N
PsNAC24	550	62717.07	8.37	45.96	65.73	-0.584	N
PsNAC25	343	38498.15	5.20	51.01	60.82	-0.951	N
PsNAC26	258	29559.64	4.47	53.59	68.06	-0.660	N
PsNAC27	462	52324.04	5.49	47.53	60.97	-0.594	N
PsNAC28	493	55846.54	4.92	49.91	68.95	-0.721	N
PsNAC29	228	25772.05	6.24	41.29	59.87	-0.724	Ch, Cy
PsNAC30	256	28720.21	9.12	44.88	76.56	-0.435	N
PsNAC31	190	22464.45	9.18	32.46	55.95	-0.896	N, Cy, M
PsNAC32	296	33807.25	8.34	44.13	69.86	-0.603	N
PsNAC33	411	46061.36	7.00	49.40	60.78	-0.664	N
PsNAC34	232	26910.10	9.58	34.04	81.12	-0.506	M
PsNAC35	346	39163.57	5.01	49.79	60.90	-0.637	Cy, N
PsNAC36	406	46861.95	5.15	46.13	67.49	-0.749	N
PsNAC37	226	26265.15	6.25	40.55	75.93	-0.410	Cy
PsNAC38	273	31176.35	6.15	27.10	67.58	-0.526	Cy
PsNAC39	481	53093.93	6.80	53.06	61.66	-0.611	N
PsNAC40	289	32722.66	9.42	48.65	72.21	-0.454	N

Gene name	Number of amino acids	Molecular weight (kDa)	Isoelectric point	Instability index	Aliphatic index	Grand average of hydropathicity	Subcellular localization
PsNAC41	289	33259.88	6.34	45.33	70.52	-0.610	N
PsNAC42	173	20212.17	9.71	39.14	54.68	-0.888	M, Cy
PsNAC43	303	34706.95	9.55	44.27	62.48	-0.741	N
PsNAC44	162	18939.61	8.49	47.74	69.81	-0.596	Cy
PsNAC45	135	15344.43	6.72	40.73	64.37	-0.559	Cy, N
PsNAC46	311	35833.72	8.77	47.58	56.11	-0.763	N, Cy
PsNAC47	215	25282.93	5.48	48.75	53.95	-1.055	N
PsNAC48	265	30405.41	8.76	32.21	60.38	-0.808	Ch, Cy, N

Note: N, nuclear; M, mitochondrial; Cy, cytoplasmic; Ch, chloroplast; E, extracellular.
