# Supplementary material for: Genome-Wide Identification of NAC Gene Family Members of Tree Peony (Paeonia suffruticosa Andrews) and Their Expression under Heat and Waterlogging Stress
Source: Int J Mol Sci. 2024 Aug 28;25(17):9312. doi: 10.3390/ijms25179312 (PMC11395581; doi:10.3390/ijms25179312)
Supplement: Supplementary file 1 [file ijms-25-09312-s001.zip › Table S1.pdf]

Table S1. Primer sequences of genes used for qRT-PCR analysis

Gene name	Forward primer sequence (5'-3')	Reverse primer sequence (5'-3')	Amplicon size (bp)
Ubiquitin	GACCTATACCAAGCCGAAG	CGTTCCAGCACCACAATC	142
PsNAC06	GGGTACTGGAAGGCTTCTGG	CTGCAGCATCTTCCCTTTCG	145
PsNAC23	AGTCTGTGACTCCCCAACCT	TTCTACGCACTCCAACTCGG	122
PsNAC37	GATCATGCACGAGTTTCGGC	AGCTGTGGTGCCATTCTCTC	105
PsNAC38	CAGATGCTAACTTGCGCTGG	ATGGCCAAGAATCTCCGGTG	180
PsNAC41	CGTGGTGGCAAAACTGACTG	TCTTCCATTGCTGCCCTCTG	140
PsNAC47	GCATTTGACAGACGGCCATC	GCGAGCTTGTTCTCTCCACT	127
